# Medical Microscopy

**Published:** 1893-06

**Authors:** 


*Medical Microscopy.* By FRANK J. WETHERED, M.D. Pp. xx., 412. London: H. K. LEWIS. 1892.

We have no desire to elevate microscopy into a speciality. The apparatus and methods are needful in every department of science, and to the medical student are indispensable. Every book on clinical diagnosis must deal with the results of microscopic investigations in almost every chapter or page, but year by year the methods are becoming more numerous and more important, and the microscope is now an absolute necessity for accurate diagnosis, not only in renal but in thoracic and other diseases. The student should get to think that his microscope is a necessary part of himself whenever he is called on for clinical work, and during the first few months he does need a special text-book which will teach him at once what to do and how to do it.

Without this he wastes much time in trying various useless methods, and becomes disheartened by constant failures to find the objects for which he is looking, but which he misses in consequence of defects in his mode of working. The experience of others who have preceded him will save much useless work and many a disappointment.

This book is well designed, well printed, abundantly illustrated, and well up to date. It includes the elements of bacteriological methods, and gives the formulæ for the various aniline stains needful for clinical work. It may well take the place of older and better known text-books of microscopy, and we strongly advise all students to purchase it and use it.

